# Belief in Unconscious Repressed Memory Persists

**DOI:** 10.1177/1745691621990628

**Published:** 2021-03-12

**Authors:** Henry Otgaar, Mark L. Howe, Olivier Dodier, Scott O. Lilienfeld, Elizabeth F. Loftus, Steven Jay Lynn, Harald Merckelbach, Lawrence Patihis

**Affiliations:** 1 Faculty of Psychology and Neuroscience, Section of Forensic Psychology, Maastricht University; 2 Department of Psychology, City, University of London; 3 Leuvens Institute for Criminology, Faculty of Law, KU Leuven; 4 Department of Psychology, University of Nantes, France; 5 Department of Psychology, Emory University; 6 Department of Psychological Science, University of California, Irvine; 7 Laboratory of Consciousness, Cognition, and Psychopathology, Binghamton University; 8 Department of Psychology, University of Portsmouth

**Keywords:** repressed memory, memory wars, unconscious, false memory

## Abstract

On the basis of converging research, we concluded that the controversial topic of unconscious blockage of psychological trauma (i.e., repressed memory) remains very much alive in clinical, legal, and academic contexts. In his commentary, Brewin (this issue, p. 443) conducted a cocitation analysis and concluded that scholars do not adhere to the concept of unconscious repression. Furthermore, he argued that previous survey research did not specifically assess *unconscious* repression. Here, we present critical evidence that runs counter to his claims. First, we inspected his cocitation analysis and found that some scholars support notions that are closely related to unconscious repression. Furthermore, we conducted another analysis on the basis of articles’ similarity. Again, we found examples of scholars specifically endorsing unconscious repressed memories. Second, as opposed to what Brewin reports, recent survey research now exists that bears directly on people’s beliefs regarding unconscious repression. This work reveals that large percentages of people (e.g., students and eye-movement desensitization and reprocessing [EMDR] clinicians) endorse the concept of unconscious repressed memories. The belief in unconscious repressed memory can continue to contribute to harmful consequences in clinical, legal, and academic domains (e.g., false accusations of abuse).

In a previous review, we (Otgaar et al., 2019) concluded that the controversial issue of unconscious blockage of psychological trauma or repressed memory remains very much alive in clinical, legal, and academic contexts. In response to our claim, Brewin (2021) offered evidence that he argued is “incompatible” (p. 449) with our conclusions. For example, Brewin claimed that few if any scholars refer to unconscious repression. In addition, he asserted that survey research on repressed memories does not assess unconscious repression. Here, we present several lines of evidence indicating that the topic of repressed memory persists.

As Holmes (1994) anticipated, the terminology used to describe repressed memories has changed and broadened greatly over time; theorists and researchers have used a variety of terms (e.g., dissociation, dissociative amnesia, engrams, and body memories) as substitutes for the monolithic and often vaguely defined term “repressed memory” to refer to the unconscious banishment of memories from consciousness. The terms listed do not necessarily convey whether “repressed memories” refer to the process or outcome of unconscious repression. Nevertheless, we suggest that the general construct of unconscious repressed memories can be encompassed by diverse hypotheses and claims regarding memory, even though the exact term “repressed memory” is not invoked.

## What Do Scholars Mean by Repressed Memory?

Recently, we presented evidence that many people in clinical, legal, and academic fields continue to believe in repressed memories (Otgaar et al., 2019). This belief lay at the heart of the so-called “memory wars” of the 1990s (Loftus & Ketcham, 1994). On the basis of converging research, we argued that the memory wars still endure in multiple quarters. According to repressed-memory proponents, repression can involve the automatic and unconscious blockage of autobiographical experiences of trauma (e.g., sexual abuse). Furthermore, unconscious repressed memories are said to lead to physical and mental health problems, and recovery of the repressed memory is crucial to symptom relief (e.g., van der Kolk & Fisler, 1995). As we observed (Otgaar et al., 2019), the scientific support for unconscious repressed memories is weak or even nonexistent. In this respect, we find it encouraging that Brewin similarly appears to express skepticism regarding unconscious repression (see also Brewin & Andrews, 2014). Apart from plausible alternative explanations for people not remembering trauma (e.g., encoding failures, ordinary forgetting, reinterpretation of traumatic experiences), a wealth of research demonstrates that traumatic experiences are not repressed but actually well remembered (e.g., McNally, 2005).

Brewin conducted a cocitation analysis to examine the major publications agreeing with the concept of repression. He argued that none of the articles detected in this analysis supported the controversial unconscious version of repression. He proceeded to conclude that, in contrast to what we proposed, scholars do not endorse this unconscious variant of repression. However, there are several problems with Brewin’s analysis. First, although Brewin stated that none of the sources endorsed the unconscious variant of repression, this contention cannot be verified with the information he presented. More specifically, he presented only a table with the author names of books and articles without describing their content (e.g., writings on conscious suppression). Second, when we inspected the content of these sources, we found clear evidence of references to unconscious repression and problematic assumptions related to the construct.^1^ For example, Herman and Schatzow (1987) wrote that

Patients were categorized as having severe memory deficits if they could recall very little from childhood, if they reported recent eruption into consciousness of memories that had been entirely repressed, or if this kind of recall occurred during the course of group treatment. (p. 4)

These authors endorse the idea of unconscious repression (e.g., “eruption into consciousness of memories that had been entirely repressed”). Likewise, although we agree with Brewin (2021) that “clinical evidence” (p. 443) shows that there are many types of memories elicited without suggestion, “clinical evidence” is no guarantee that recovered memories are true.

To give another example, Terr (1991) argued that “spontaneous dissociation” could underlie “amnesia for certain periods of childhood life” (p. 330).

Here, one might quibble with whether repression is isomorphic with dissociation. Yet the idea of “spontaneous dissociation” is arguably indistinguishable from unconscious repression, in which large blocks of experience are banned from memory. In addition, contrary to Brewin’s assertions, the notion of unconscious repression remains accepted by many scholars under the guise of dissociative amnesia in the influential fifth edition of the *Diagnostic and Statistical Manual of Mental Disorders* (American Psychiatric Association, 2013; *DSM–5*). According to the *DSM–5*, dissociative amnesia “involves a period of time when there is an inability to recall important biographical information” (p. 298) and is “always potentially reversible because the memory has been successfully stored (p. 298). (These quotes appeared in our original article [Otgaar et al., 2019] as well.) These statements again imply the unconscious version of repression. The inclusion of dissociative amnesia in the *DSM–5*—so tightly aligned with the concept of unconscious repression—may be one of the most important reasons why this debate is likely to extend for years to come.

**Fig. 1.**
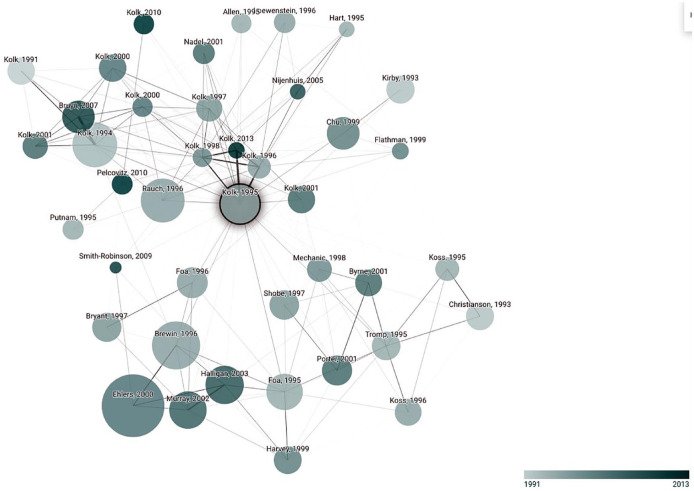
Graph of papers arranged according to their similarity. Darker colors represent more recent articles; lighter colors represent older articles. Hart = van der Hart; Kolk = Van der Kolk; Minnen = Van Minnen.

Furthermore, a limitation of cocitation analysis (as used in Brewin, 2021) is that it includes only sources that cite each other. An alternative analysis, using the website https://www.connectedpapers.com, not only takes advantage of the principles of cocitation and bibliographic coupling but also rearranges sources according to their similarity. The benefit of such an analysis is that it can encompass more sources related to unconscious repression. To conduct such an analysis, an article identifier (e.g., DOI number, title) needs to be inserted, after which a graph is created in which articles are visually displayed in terms of their similarity to the source in question. Our strategy was to include an identifier of an article that contains problematic assumptions concerning repressed memory. We elected to use the widely cited (over 1,700 citations as of this writing according to the *Google Scholar* database) article by Van der Kolk and Fisler (1995), which maintained that repressed memories of trauma can exert a physical toll. According to this body-keeps-the-score hypothesis, trauma can be “entirely organized on an implicit or perceptual level, without an accompanying narrative about what happened” (Van der Kolk & Fisler, 1995, p. 512). We selected this article given that we used it prominently in our previous article as an example of the prevalence of the idea of unconscious repressed memory (Otgaar et al., 2019; see also McNally, 2005). Figure 1 displays a visual representation of articles that share similarities with Van der Kolk and Fisler. We inspected these papers and, in contrast to Brewin, found clear indications that scholars referred to unconscious repression. For example, Van der Hart and Nijenhuis (1995)^2^ wrote that memory loss due to trauma “involves a reversible memory impairment in which memories of personal experience cannot be retrieved in a verbal form, or, if temporarily retrieved, cannot be wholly retained in consciousness” (p. 1).

Another example: Van der Kolk et al. (2001) postulated that “traumatic memories persist primarily as implicit, behavioral, and somatic memories” (p. 24). There are also examples of scholarly mention of unconscious repression that were not part of our analysis. For example, Axmacher et al. (2010) argued that there are

two problematic cases involving extremely negative emotions: the emergence of an unconscious conflict, which is subject to *repression*, and traumatic events that overstress a person’s executive capabilities and thus lead to *dissociation*. As a result, conscious recall of these contents is impaired, but they continue to exert an unconscious effect which dramatically influences subsequent life—for example, by uncontrollably occurring intrusions and dissociative flashbacks, panic attacks, or psychosomatic symptoms. (para. 4)

In sharp contrast with Brewin, we find substantial evidence to the contrary and further contend that the concept is very much alive and is likely to endure in the future. Even though the terminology has seemingly changed over time (from *repressed memory* to *dissociation* and *dissociative amnesia*), the idea persists that a person can be physically or sexually abused, somehow unconsciously extirpate the disturbing experience from memory, and then recall it year later in pristine detail. Problematically, scholars and clinicians who endorse a belief in unconscious repression might advocate for associated and problematic practices, such as attempting to unearth repressed traumatic memories using suggestive procedures in psychotherapy (Loftus & Ketcham, 1994).

## Surveys on Unconscious Repressed Memory

Many researchers have surveyed people from the general public, clinicians, students, and legal professionals regarding their beliefs in repressed memories (e.g., Magnussen & Melinder, 2012; Ost et al., 2017; Patihis et al., 2014). Combining data from all surveys, we reported that the belief in repressed memories is widespread (58%; *n* = 4,745; Otgaar et al., 2019). Brewin criticized this research because it relied on a single questionnaire item to assess repressed memory (e.g., “Traumatic memories can be repressed for many years and then recovered”; Kassin et al., 2001) that does not refer specifically to *unconscious* repression.

There are several flaws in his criticism. First, several researchers have explicitly asked participants about their belief in *unconscious* repressed memory. For example, Houben and colleagues (2019) asked therapists who practice the widely used technique of eye-movement desensitization and reprocessing (EMDR) whether “the mind is capable of unconsciously blocking out memories of traumatic events.” EMDR is a highly popular intervention for posttraumatic stress disorder (Cuijpers et al., 2020). In two studies, Houben and colleagues showed that large percentages of small samples of EMDR therapists agreed with this statement (Study 1: 91.6%, 11/12; Study 2: 70.7%, 29/41).

Second, Brewin referred to recent work that he and colleagues published (Brewin et al., 2019) asking participants to respond to two items concerning repressed memory, one that has frequently been used in previous work (“Traumatic memories can be repressed for many years and then recovered”) and one focusing on *conscious* repression (“Traumatic experiences can be deliberately blocked out for many years and then recovered”). Participants agreed to these statements at similar rates. Brewin and colleagues argued that one explanation for this finding was that participants actually endorse a belief in *conscious* repression. In a recent study, we commented on Brewin et al.’s research as they did not specifically ask participants about their beliefs in *unconscious* repression (Otgaar, Wang, Howe, et al., 2020). We corrected this shortcoming and also surveyed participants (Study 1: *N* = 230; Study 2: *N* = 79) about their belief in unconscious repression. We found that many people endorse this belief (Study 1: 59.2%; 45/86; Study 2: 67.1%; 53/79).

Furthermore, to examine more specific beliefs concerning the issue of repressed memory (e.g., whether repressed memories can lead to psychopathological symptoms), we also surveyed people from the (French) general public (*N* = 1,125) and provided them with more specific statements related to the topic of repressed memories. It is important to note that we presented them with statements such as “Unconscious repressed memories can cause mental health problems (e.g., depressive symptoms),” “Unconscious repressed memories are memories of events that people are unaware that they happened to them,” “Unconscious repressed memories can be retrieved in therapy accurately,” and “When unconscious repressed traumatic memories cause mental health problems, it is necessary to recover the repressed memories to heal.”

Participants had to rate these statements on a 6-point Likert scale (1 = *strongly disagree*, 2 = *disagree*, 3 = *slightly disagree*, 4 = *slightly agree*, 5 = *agree*, 6 = *strongly agree*). We found that 91.6% (934/1020) of the participants agreed (i.e., provided ratings of 4, 5, or 6) that repressed memories can lead to mental health problems. Furthermore, we found that 51.9% (523/1008) of participants agreed with the statement that repressed memories concern memories of events that people are unaware of. In addition, 55.6% (567/1020) of participants indicated that repressed memories can be accurately retrieved in therapy. Finally, 66% (661/1003) of the participants endorsed the idea that the recovery of repressed memories is needed to heal (for more information, including demographics, see https://osf.io/n9fbg/).

Third, in another recent study, we asked follow-up questions about what people mean when they endorse repressed memory (Otgaar, Wang, Dodier, et al., 2020; see https://osf.io/puzdy/). Specifically, we asked participants whether “traumatic memories are often repressed.” If people agreed with this item, they received additional questions that checked whether they meant those traumatic memories are (a) accessible during repression and (b) unconscious during repression. We found that 89.5% (*n* = 909) agreed to some extent that traumatic memories can be repressed and, of those, 73.7% (*n* = 670) agreed that such memories are inaccessible and 80.9% (*n* = 735) agreed that such memories are unconscious. Both follow-up questions’ results are strongly consistent with the controversial concept of unconscious repression.

Taken together, these data show that many people believe in (unconscious) repressed memory. Furthermore, high percentages of students and perhaps clinicians—at least those who use EMDR—endorse notions highly consistent with unconscious repression. These beliefs lie at the heart of the memory wars and are strongly consistent with our conclusion that these wars are far from over (see also Otgaar et al., 2019).

## Memory Suppression and False Memories

We have shown that, contrary to Brewin’s assertions, many scholars continue to refer to unconscious repression. Furthermore, large percentages of people endorse this concept (e.g., Otgaar, Wang, Howe, et al., 2020). Apart from problems concerning the notion of unconscious repression, it is also important to discuss memory phenomena related to unconscious repression (i.e., memory suppression) and the memory wars (i.e., false memories).

Specifically, a critical prong in the memory wars concerns the controversial idea that trauma can unconsciously block autobiographical experiences. However, an alternative variant of repression presumably happens through *conscious* control and is sometimes referred to as *memory suppression* or *motivated forgetting* (e.g., Anderson & Green, 2001). Brewin, Li, et al. (2020) argued that memory suppression serves as a likely candidate for the forgetting of autobiographical memories and their recovery after many years. Nevertheless, no research has convincingly demonstrated memory suppression for autobiographical experiences in the laboratory (Otgaar et al., 2019), let alone for years or decades in everyday life. Furthermore, recent research suggests that memory suppression has been difficult to replicate (e.g., Bulevich et al., 2006). Adding to these doubts, Wessel et al. (2020) conducted a multiverse analysis^3^ on several memory-suppression experiments and failed to find evidence for consistent suppression effects. They concluded that their analysis “raises problems for inhibition theory and its implication that repression is a viable mechanism of forgetting” (p. 870).

Another important aspect of the memory wars is that legal cases have revealed that, in certain cases, the “recovery” of false memories of childhood abuse was the by-product of therapist suggestions that clients had repressed such memories (Loftus, 1993, 1994).^4^ A consequence of these legal cases was that memory researchers started to examine the conditions under which people could create autobiographical false memories. Loftus and Pickrell (1995) were among the first to show that people can be led to falsely believe and remember an autobiographical event that never happened.

Brewin, Andrews, & Mickes (2020) criticized research using such paradigms by suggesting that only a “small minority” (p. 123) of participants are susceptible to false-memory implantation. However, a recent review of studies of false-memory implantation showed that when transcripts of these studies were scored using a detailed coding scheme, 30.4% were classified as false memories and another 23% were classified as accepting the false event (Scoboria et al., 2017). These percentages combined are surely not a small minority. Even setting aside these high percentages and the point that different implantation procedures might have led to different percentages, the crucial take-home message from memory implantation studies is that it *is* possible to make people falsely remember a nonexperienced autobiographical event (Nash et al., 2017; Smeets et al., 2017). Furthermore, recent research shows that false-memory implantation occurs at similar rates for repeated and single occurring events (Calado et al., 2020).

We are in agreement with Brewin that we must be careful (a) not to discredit genuine cases of sexual trauma and (b) to take corroborated claims of such trauma seriously. Nevertheless, what are the dangers of dismissing evidence demonstrating that the memory wars are still being fought? A case in point concerns the time within which sexual-abuse crimes can be prosecuted, also called the statute of limitation period. Recently, several European countries have extended or even abolished these limitation periods on the basis of the premise that repressed memories exist (for an example of extension in France, see Dodier & Tomas, 2019). The rationale is that because traumatic experiences can make people unconsciously forget the experience for decades, they cannot know of the crime until the “memory” is recovered in therapy or in everyday life. Therefore, because of unconscious repression, the statute of limitations cannot begin at the time of the alleged abuse or at a starting point set out in the law (e.g., 18th birthday), but must instead begin when the memory of that abuse is recovered. An adverse side effect of the removal of these limits or extensions of limitation periods is that they may pave the way for therapeutically induced false recovered memories of abuse and consequent miscarriages of justice.

## Concluding Remarks

We have shown, contrary to Brewin’s assertions, that (a) some major scholars, including contemporary authors, do continue to refer to the controversial phenomenon of unconscious repression and (b) large proportions of people, including students and EMDR clinicians, endorse unconscious repression (likely in the forms of the Freudian version, the *DSM* diagnosis of dissociative amnesia, or both). Such evidence suggests that the memory wars are far from over. The ongoing belief in unconscious repression (but also its ostensible conscious form) can be harmful in multiple contexts. For example, undergraduate and graduate psychology students, informed that there is good evidence for unconscious repression, may be inclined to use suggestive techniques to excavate purported repressed memories of abuse when they become practicing clinicians. A second example: Legal professionals (e.g., judges) who believe in unconscious repression may uncritically accept the claims of alleged victims of abuse reporting dissociative amnesia, in turn contributing to wrongful convictions. We hope that Brewin would agree with us that it is worthwhile to inform students, clinicians, and legal professionals about research that casts doubt on this phenomenon, including studies of retractors (i.e., people who have repudiated earlier claims of being abused; e.g., de Rivera, 2000). Furthermore, we should draw their attention to case and experimental studies of how certain therapeutic techniques may inadvertently create false memories (e.g., Houben et al., 2020).

In one article, Brewin, Andrews, and Mickes (2020) warned of the dangers of “overenthusiastically championing conclusions based on limited data” (p. 125). We agree. Nevertheless, it can be at least equally dangerous to omit conclusions based on *available* data that reveals that the idea of unconscious repression continues to be accepted among many scholars and laypersons.

## Transparency

*Action Editor:* Laura A. King

*Editor:* Laura A. King

## Notes

1. We identified other references to unconscious repression and problematic assumptions related to repressed memory. For the sake of brevity, we have not included them in the manuscript text, but they can be accessed at https://osf.io/2yck8/

2. In the graph, this article was designated as “Nijenhuis (2005)” but refers to Van der Hart and Nijenhuis (1995).

3. A multiverse analysis examines all possible and plausible analyses (i.e., universes) that exist for testing a certain hypothesis

4. For a legal case on therapist-induced false memories, see “Shrink implanted false memories” (2012).
